# Impact of Nursing Interventions via Telephone and Email on the Quality of Life of Patients with Inflammatory Bowel Disease: Preliminary Results of a Comparative Observational Study

**DOI:** 10.3390/healthcare12242538

**Published:** 2024-12-16

**Authors:** Caterina Mercuri, Vincenza Giordano, Vincenzo Bosco, Nicola Serra, Rocco Spagnuolo, Rita Nocerino, Teresa Rea, Carmen Colaci, Assunta Guillari, Patrizia Doldo, Silvio Simeone

**Affiliations:** 1 Department of Clinical and Experimental Medicine, University of Catanzaro MagnaGraecia, 88100 Catanzaro, Italy; c.mercuri@unicz.it (C.M.); carmen.colaci@studenti.unicz.it (C.C.); doldo@unicz.it (P.D.); silvio.simeone@unicz.it (S.S.); 2 Cardarelli Hospital, 80131 Naples, Italy; enza-giordano@hotmail.it; 3 Department of Medical and Surgical Sciences, University Hospital Mater Domini, Magna Graecia University, 88100 Catanzaro, Italy; vincenzo.bosco@unicz.it; 4 Department of Neuroscience, Reproductive Sciences and Dentistry-Audiology Section, University of Naples Federico II, Via Pansini 5, 80131 Naples, Italy; nicola.serra5@gmail.com; 5 Department of Health Sciences, University “Magna Graecia”, 88100 Catanzaro, Italy; spagnuolo@unicz.it; 6 Department of Translational Medical Science, University of Naples “Federico II”, 80131 Naples, Italy; assunta.guillari@unina.it; 7 ImmunoNutritionLab at CEINGE—Advanced Biotechnologies, University of Naples “Federico II”, 80131 Naples, Italy; 8 Department of Public Health, University of Naples “Federico II”, 80131 Naples, Italy; teresa.rea@unina.it

**Keywords:** symptoms, quality of life, remote nurse intervention, telephone, email

## Abstract

Background: Inflammatory bowel disease (IBD), encompassing ulcerative colitis and Crohn’s disease, is a heterogeneous chronic condition characterized by periods of relapse and remission. Ulcerative colitis involves inflammation of the colon and rectum mucosa, while Crohn’s disease causes deeper, transmural inflammation affecting all four gut layers from the mouth to the anus and can lead to complications such as fistulation. IBD significantly impacts patients’ physical and psychological well-being, thus reducing their quality of life (QoL). We aimed to evaluate the effectiveness of nursing intervention facilitated through telephone and email support in improving the quality of life (QoL) of Inflammatory Bowel Disease (IBD) patients. Methods: A pilot comparative observational design with pre-test and post-test assessments was employed, involving 50 participants assigned to either an intervention group (Group A, n = 26) or a control group (Group B, n = 24). Group A received regular telephone consultations and prompt email responses from trained nurses; Group B received standard care. Data were collected at baseline and six months post-intervention (T1) using the Patient-Reported Outcomes Measurement Information System (PROMIS^®^) and Pittsburgh Sleep Quality Index. Results: Group A showed significant improvements in anxiety, depression, fatigue, and sleep quality, with *p*-values indicating the significance of these findings. Conclusions: Tailored nursing support via remote communication significantly benefits IBD patients by alleviating psychological distress and enhancing their overall well-being, underscoring the importance of integrating such interventions into standard IBD care practices.

## 1. Introduction

Inflammatory bowel disease (IBD), which includes ulcerative colitis (UC) and Crohn’s disease (CD), is a chronic condition characterized by inflammation of the intestinal mucosa, the causes of which are still unclear [1,2]. UC causes continuous inflammation that affects only the mucosa of the colon, whereas CD is defined by inflammation that crosses the entire thickness of the intestinal wall and by the presence of scattered lesions throughout the gastrointestinal tract [3,4].

The prevalence and incidence of IBD, characterized by persistent and recurrent symptoms that are difficult to treat, are increasing worldwide [2,5,6]. Patients with IBD often experience symptoms such as abdominal pain and diarrhea, which can lead to psychological problems such as anxiety and depression, negatively affecting both sleep quality and physical and mental health [7,8,9,10,11]. Even during the remission phase, due to brain–gut interaction, these symptoms persist [12]. Psychological factors can trigger or modify the clinical presentation of IBD symptoms [13]. Furthermore, the bidirectional relationship between IBD and psychological problems can be explained by the gut–brain axis, whereby anxiety and depressive symptoms or disorders may increase gut inflammation, contributing to disease relapse, while gut inflammation may negatively influence mood [14]. IBD significantly reduces patients’ QoL due to its physical and psychological impact [15]. Psychological disorders are considered independent predictors of symptom severity in IBD, contributing to a reduction in QoL and an increase in health service utilization and caregiver burden [16,17,18,19,20]. A recent systematic review found that the prevalence of anxiety symptoms in patients with IBD was 32.1%, while that of depressive symptoms was 25.2% [21]. There is a close link between anxiety, depression, sleep disturbances, and low QoL both at the onset and during the clinical course of IBD [21,22,23,24,25]. Fatigue associated with IBD also negatively impacts health-related QoL and daily activities, as evidenced by several studies [26,27,28,29,30,31]. Similarly, digestive symptoms significantly affect patients’ QoL, leading to disruptions in daily activities, work productivity, and social interactions [32,33]. The impact of illness on work results in absenteeism, reduced working hours, and potential changes in career choice, increasing the financial burden and reducing life satisfaction [34,35,36,37]. Work and social life are also impacted by various forms of stigma, which affect the perceived QoL of people with IBD, especially in social and psychological dimensions [38,39]. Stigma can have considerable repercussions on the QoL of people with IBD and can even worsen the clinical signs and symptoms of the disease [40]. Studies show a clear correlation between adverse psychological conditions and unfavorable clinical and psychosocial outcomes in people with IBD [41,42].

Among the professions involved in the management of IBD, nurses play an important role. Continuity of care and access to outpatient clinics, where IBD nurses work, are key points emphasized by the European Organization for Crohn’s and Colitis [43]. Although the nursing role in IBD is generally considered beneficial to patients, it remains ill-defined [44]. There is no established national or international consensus regarding the level of education needed for nurses engaged in this specific area. However, experienced IBD nurses often take a central and autonomous role, providing consultation, coordinating care, and participating in treatment planning and evaluation [44] in collaboration with other specialists, such as gastroenterologists [45].

Several authors have highlighted the importance of the role of nurses in IBD, emphasizing their ability to provide counseling, education, and physical and emotional support to patients, as well as to facilitate access to services through channels such as telephone and email [46]. Recent studies further indicate that the active involvement of nurses helps reduce hospitalizations and emergency room visits through timely and targeted interventions, thereby reducing overall healthcare costs [47,48]. Their presence in multidisciplinary teams ensures continuous follow-up while enhancing treatment adherence, symptom management, and emotional support for patients [47,48]. Indeed, the availability of telephone access for patients served by IBD nurses has been associated with positive outcomes [48,49]. Despite the limited number of specific studies on the role of nurses in IBD and how they provide follow-up, some authors have shown that nurse-led follow-up can reduce hospitalizations, shorten the time to recurrence, improve understanding of the disease and treatments, increase patient satisfaction, and reduce healthcare costs [47,48,50,51,52]. Furthermore, nurse-managed interventions, such as telephone consultations or follow-up meetings, have been associated with significant reductions in healthcare costs and overall improvements in disease management [53]. Consequently, more research is needed on the role of nurses in promoting the QoL of IBD patients [45]. In Italy, to the best of our knowledge, only two studies on IBD have been conducted about QoL and IBD: the study by Mancina et al. [10], investigating the influence of symptoms on the QoL in patients with IBD, and the study by Vegni et al. [25], exploring how the perception of the disease can contribute to improving the management of patients with IBD. However, none of the studies in Italy investigated the effects of nursing interventions in patients with IBD. Therefore, the purpose of this study is to examine the impact of a nursing intervention using telephone calls and emailing on the QoL of patients with IBD. The research question guiding this study is: ‘Does a tailored nursing intervention improve QoL and the psychological and physical well-being of IBD patients compared to standard care?’ To address this question, we hypothesize that: (1) patients receiving the nursing intervention will show significant improvements in anxiety, depression, fatigue, and sleep quality compared to the control group; and (2) the intervention group will demonstrate a greater increase in overall QoL at six months compared to baseline values and to the control group.

## 2. Materials and Methods

### 2.1. Study Design and Setting

This study was a comparative observational study conducted from September 2023 to March 2024.

The study assessed the effectiveness of an intervention that established a direct channel between the referring nurse of the Gastroenterology and Operative Endoscopy Clinic of the Magna Graecia University of Catanzaro (Italy) and the patients with baseline and 6-month follow-ups.

This intervention was facilitated through telephone and a dedicated email address. Nurses provided consultations, emotional support, and practical advice on disease management. This study adhered to the Transparent Reporting of Evaluations with Nonrandomized Designs (TREND) guidelines.

### 2.2. Participants

The sample consisted of patients with Inflammatory Bowel Diseases (IBDs) who consecutively attended the Gastroenterology and Operative Endoscopy Clinic of the Magna Graecia University of Catanzaro. The enrollment, sequential allocation, explanation of the intervention, and administration of the questionnaires were conducted by the nursing staff. In Group A, patients received the nursing intervention, while Group B served as the control group.

### 2.3. Inclusion and Exclusion Criteria

The inclusion criteria for the sample were as follows: (a) confirmed diagnosis of IBD; (b) patients aged 18–75 years; (c) proficiency in speaking and understanding Italian; (d) access to the internet; (e) knowledge of how to use the telephone and/or mobile phone correctly; and (f) willingness to participate in the study. Exclusion criteria for the sample included: (a) presence of other chronic diseases; (b) severe organ dysfunction; and (c) cognitive alterations.

### 2.4. Nursing Intervention

Group A received a nursing intervention that included direct access to a telephone and email service. Qualified nurses supported the patients through regular telephone consultations and prompt responses to patient questions and concerns sent via email. The intervention was structured to meet the individual needs of the patients and provided information about IBD, emotional support, and practical advice on lifestyle, disease management, and daily medication therapy. Additionally, patients received specific suggestions to improve medication adherence and appointment management through telephone and postal consultations. The support provided to Group A consisted of a structured intervention delivered by specialist nurses through remote communication channels, including telephone and email. Each patient in Group A received a scheduled phone call every two weeks, totaling 12 calls over the six-month study period. Additionally, patients could request extra calls for urgent clarification or specific support as needed. Each call lasted approximately 20–30 min, tailored to the individual needs of the patient. The intervention addressed multiple aspects of care. Nurses offered guidance on symptom management, providing practical advice for handling common issues such as diarrhea, abdominal pain, and fatigue. They also focused on improving medication adherence by sharing strategies and reminders to help patients follow their treatment plans and manage medications effectively. Nurses teaching relaxation techniques to alleviate anxiety and offering advice to enhance sleep quality. Lifestyle guidance was another crucial component, encompassing stress management, meal planning, and recommendations for physical activity. Patients were also assisted in preparing for clinical appointments, including advice on formulating questions and interpreting diagnostic test results. Email communication complemented the telephone support, primarily serving to address specific patient inquiries or provide additional information following phone calls. Nurses ensured that responses were delivered within 24 h, offering timely and continuous support throughout the intervention.

The control, Group B, did not undergo this intervention. Instead, they received the usual standard of care, including regular scheduled follow-ups, urgent follow-ups as needed, and the possibility of receiving additional information and support, if necessary, upon specific request through normal communication channels.

Interventions were provided both in response to patient-initiated requests and as scheduled follow-ups. Nurses did not assist patients in completing questionnaires, which were filled out independently.

### 2.5. Data Collection Tools

Participants were asked to complete an ad hoc questionnaire. The questionnaire was administered in paper format, delivered into the hands of each enrolled patient. The questionnaire was administered to participants at two time points: baseline (T0) and six months post-intervention (T1). At baseline, participants completed the questionnaire independently before receiving any intervention. The administration process ensured that the questionnaires were self-administered, with no assistance from nurses or researchers, to avoid bias and maintain the integrity of the responses. This methodology was consistent across both the intervention group and the control group, ensuring comparability of the data collected at both time points. The questionnaire covered socio-demographic information and health-related QoL measures. At the six-month follow-up, participants completed the same set of questionnaires to assess changes over time. In detail, the Patient-Reported Outcomes Measurement Information System (PROMIS^®^) is a multidimensional assessment tool designed to measure various aspects of patients’ physical, mental, and social well-being [54,55]. It was developed by the US National Institutes of Health (NIH) and provides a set of standardized questionnaires to assess different dimensions of health [56,57]. In this study, the PROMIS^®^ questionnaire was used to assess the presence of clinically significant symptoms of depression, anxiety, fatigue, satisfaction with social participation, and overall well-being [56,58,59,60]. The scores obtained from the questionnaires were dichotomized into “yes” and “no” using a T-score cut-off of 50. T-scores of 50 or greater indicated the presence of a clinically significant symptom, while T-scores less than 50 indicated the absence of a clinically significant symptom [10,61,62].

The Pittsburgh Sleep Quality Index (PSQI) was used to investigate sleep quality perception, sleep habits, sleep problems, and sleep-related disturbances [63]. It has been validated for use in Italy [64]. The PSQI is a self-administered questionnaire consisting of 19 items with different response formats, including 5-point Likert scales and open-ended responses. The PSQI measures seven key dimensions of sleep quality:

- Subjective sleep quality: assesses the subject’s general perception of sleep quality;
- Sleep latency: indicates the time taken to fall asleep;
- Sleep duration: represents the total time spent sleeping during the night;
- Habitual Sleep Efficiency: calculates the percentage of time spent asleep compared to time spent in bed;
- Sleep disorders: assesses the presence and frequency of sleep disorders such as sleep apnea, snoring, excessive daytime sleepiness;
- Sleep medication use: assesses the frequency of sleep medication use;
- Daytime dysfunction: measures the degree of drowsiness or difficulty performing tasks during the day due to the quality of sleep at night.

Each dimension is scored from one to three, with higher scores indicating greater impairment in sleep quality. Dimension scores are summed to give a total score ranging from 0 to 21. A total score above 5 indicates impaired sleep quality. The Italian version of the PSQI showed good reliability (α = 0.83) in discriminating between patients with sleep disorders and healthy controls. In this study, the reliability of the scores for the seven dimensions was acceptable (α = 0.70, ω = 71).

To assess health-related quality of life in patients with inflammatory bowel disease, we used the Inflammatory Bowel Disease Questionnaire (IBDQ) [65]. The questionnaire consists of 32 questions divided into four main dimensions:

1. Bowel symptoms: severity and frequency of gastrointestinal symptoms;
2. Systemic symptoms: non-specific symptoms related to the gastrointestinal system, such as fatigue and loss of appetite;
3. Emotional function: emotional well-being, including anxiety and depression;
4. Social function: impact of the disease on social relationships and social activity.

Each question had answers ranging from 1 (indicating the worst situation) to 7 (indicating the best situation). Total scores ranged from 32 to 224, with higher scores indicating better quality of life. The IBDQ questionnaire has been widely used to assess the quality of life in patients with inflammatory bowel disease [66] and is considered the most specific and reliable assessment tool for this population [67,68]. It has also been validated for use in the Italian population [69].

### 2.6. Data Collection

During the study, participants completed questionnaires both pre-operatively (T0) and 6 months later (T1) during the post-intervention follow-up. At the beginning of the study (T0), participants were recruited and completed questionnaires related to anxiety, depression, fatigue, social participation, sleep quality, and quality of life (PROMIS^®^, PSQI, IBDQ) before receiving any type of intervention. During this phase, the researchers also collected baseline data on participants’ characteristics and parameters of interest. In the six-month (T1) follow-up, participants were re-evaluated using the same tools and questionnaires used at T0. This allowed the researchers to compare the data collected at T1 with those collected at T0 to assess any changes over time, both about the effects of the intervention and other factors.

### 2.7. Ethical Considerations

Ethical approval for this study was obtained from the Ethics Committee of the Calabria Region Centre on 21 April 2022, with n° 119. The study was conducted following the guidelines of the Declaration of Helsinki. The ethical principles of informed consent, voluntary participation, right to withdraw, confidentiality, and anonymity were respected. In the intervention group, patients were fully aware that they were receiving an additional level of care compared to the control group, and this was explicitly addressed in the informed consent process.

### 2.8. Statistical Analysis

Data were presented as numbers or percentages for categorical variables. Continuous data are expressed as the mean ± standard deviation (SD), or median with Interquartile Range (IQR = [Q1, Q3]).

For inferential analysis, the Chi-square (χ^2^) test and Fisher’s exact test were used to assess significant differences in proportions or percentages between the two groups, with Fisher’s exact test applied when the Chi-square test was not appropriate. Test for normal distribution was performed by Shapiro–Wilk test.

The unpaired *t*-test was used to test differences between two means of independent groups. Particularly, when distribution was not normal, the Mann-Whitney test was performed. The paired *t*-test was used to compare the means between pre- and post-intervention within each group. Particularly, when distribution was not normal, the Wilcoxon signed-rank test (W) was performed.

Additionally, since the sample size for each subgroup was smaller, we performed the power analysis for each statistical test using the effect size. Particularly, the effect size was computed by phi coefficient for categorical variables, by *η*^2^ and *r* for non-parametric test (Mann-Whitney test and Wilcoxon signed-rank test, respectively), and by Cohen’s *d* index (paired and unpaired *t*-test).

All tests with *p* < 0.05 were considered significant. All data were analyzed with Matlab statistical toolbox version 2008 (MathWorks, Natick, MA, USA) for 32 bits Windows.

## 3. Results

Initially, 62 participants were enrolled, but only 50 of them signed the informed consent form. These exclusions were due to the following reasons: incomplete baseline data (n = 6), withdrawal from the study before the intervention began (n = 2), and failure to complete the six-month follow-up (n = 4). These participants were excluded to ensure the integrity of the dataset and the comparability of pre- and post-intervention assessments. While the exclusion of these individuals could introduce selection bias, the baseline characteristics of the excluded participants were similar to those of the analyzed cohort, minimizing the likelihood of a systematic bias influencing the results. Nevertheless, future studies should aim to reduce attrition rates and investigate the characteristics of non-respondents in more detail to better understand their potential impact on study findings. The study involved a total of 50 participants, with 26 in Group A and 24 in Group B. The baseline general characteristics of Group A and B are presented in Table 1. The last column specifically provides a comparison between the two groups; particularly, in the last column, we report the effect size results.

Table 1 shows no significant differences between Group A and B regarding sociodemographic characteristics. Particularly, from a power analysis, we found low phi values, i.e., for all comparisons the effect size showed a trivial/small effect

Table 2 shows the results obtained for the PROMIS^®^ questionnaire, focusing on anxiety, depression, fatigue, and satisfaction with social participation.

Two analyses are presented in Table 2: the first analysis compares Groups A and B, while the last column illustrates the comparison for each group between T0 and T1.

Table 2 shows that there were no significant differences between groups B and A at T0. At T1, significant differences between group B and A were observed for anxiety (64.8 vs. 54.2, *p* = 0.0001), depression (median: 54.0 vs. 50.0, *p* = 0.0013), fatigue (54.0 vs. 50.0, *p* = 0.0010), and satisfaction (57.6 vs. 48.3, *p* < 0.0001). For group B, significant differences were observed between T0 and T1. In particular, there were significant reductions in anxiety (67.7 vs 64.8, *p* = 0.0001), depression (median: 56 vs. 54, *p* = 0.0284), and fatigue (median: 57 vs. 54, *p* = 0.0136). For group A, significant differences were observed between T0 and T1. In particular, there was a significant reduction in anxiety (65.4 vs 54.2, *p* < 0.0001), depression (54.8 vs. 50.1, *p* < 0.0001), fatigue (median: 56 vs. 50, *p* < 0.0001), and social satisfaction (56.5 vs. 48.3, *p* < 0.0001).

In terms of changes in scores between T0 and T1 within groups, Group B had significant reductions in depression (median: 56 vs. 54, *p* = 0.0284) and fatigue (median: 57 vs. 54, *p* = 0.0136). Group A showed significant reductions in anxiety (65.4 vs. 54.2, *p* < 0.001), depression (54.8 vs. 50.1, *p* < 0.0001), and fatigue (median: 56 vs 50, *p* < 0.0001) and an increase in social satisfaction (56.5 vs. 48.3, *p* < 0.0001).

From power analysis, we observed that two significant statistical tests between Group A and B could be affected by biases due to small sample sizes, particularly the comparison between Group A and B at T1 for anxiety and satisfaction.

Table 3 assesses sleep quality and quality of life. Similar to Table 2, two analyses are presented. The first analysis compares group A and B, while the last column illustrates the comparison within each group between T0 and T1.

Table 3 reveals that, at T0, there were no significant differences between groups B and A for sleep quality or quantity. At T1, significant differences were observed between groups B and A for both sleep quality (median: 4 vs. 3, *p* = 0.0013) and quality of life (median: 193 vs. 199, *p* = 0.0002). A significant decrease in sleep quality was observed between T0 and T1 in both groups: Group B (median: 5 vs. 4, *p* = 0.0052); and Group A (median: 5 vs. 3, *p* < 0.0001). For quality of life, a significant increase was found between T0 and T1 only in group A (median: 192 vs. 199, *p* < 0.0001). Looking more closely at Group A, there was a significant improvement in the mean sleep quality score from time T0 to time T1 (5.2 vs. 3.2). In group B, although a significant decrease in sleep quality was observed from time T0 to time T1 (4.9 vs. 4.3), this decrease was less pronounced than in group A. In terms of quality of life, group A showed a significantly better quality of life than group B at time T1 (199.8 vs. 193.0), as indicated by a higher mean score. Looking more closely at Group A, there was a significant improvement in the mean QoL score from time T0 to time T1 (193.2 vs. 199.8). For group B, although there were no significant differences in the mean QoL score between the two time points (191.8 vs. 193.0), the group maintained a stable level of QoL over time.

Finally, in Table 3, all significant statistical tests were confirmed by effect size in power analysis.

## 4. Discussion

Inflammatory bowel disease (IBD) is a chronic, progressive, and disabling condition characterized by chronic, uncontrolled, and relapsing inflammation of the gastrointestinal tract [70,71]. Ulcerative colitis results in persistent inflammation limited to the mucosa of the colon, whereas CD is characterized by transmural inflammation, involving all layers of the intestinal wall and the presence of segmental lesions scattered throughout the gastrointestinal tract [3,4].

IBD symptoms have a negative impact on patients’ social and daily functioning, and consequently on their psychological and social well-being [72,73]. The complex management of IBD requires a specialized, multidisciplinary approach to provide a higher level of continuous care and improve outcomes [74,75]. This study aimed to evaluate how a nursing intervention supported by telephone access or email services influences the quality of life of patients with IBD. These services were offered to patients as part of their care pathway during the study period.

The results of our study indicated that at T0, there were no significant differences between Group A and Group B regarding the general characteristics of the patients. At 6 months (T1), the analysis showed significant differences between the control group (Group B) and the intervention group (Group A) regarding the parameters studied.

The onset of IBD in older age differs from the onset in younger age. This has implications for management and treatment approaches [76]. Age has been positively associated with skills, including knowledge of the disease and the performance of self-management behaviors [77]. The average time since diagnosis of IBD in our study patients was 19 years. This did not have a significant impact on the results as there was no significant difference in age at IBD diagnosis between the two groups at baseline (T0).

Overall, the results suggested that the nursing intervention positively impacted emotional well-being and quality of life (QoL).

Examining anxiety levels, no significant differences were observed at T0 between the two groups. However, at the follow-up stage (T1), there was a significant difference between the two groups, with Group A experiencing a significant decrease in anxiety levels compared to Group B (64.8 vs. 54.2, *p* = 0.0001). This suggests that the nursing intervention program had a positive effect in reducing anxiety levels in the participants compared to the control group. This finding is consistent with a systematic review conducted by Coughtrey and Pistrang [53], which highlighted the effectiveness of remote communication interventions (e.g., telephone interventions) in reducing anxiety symptoms. A study conducted by Goodhand et al. [78] confirmed that emotions such as fear are predominant in chronic disease. Uncertainty about health conditions, prognosis, fear of surgery, and cancer risk are all contributing factors to the onset of anxiety. Therefore, the use of remote technological tools enables patients to actively participate in managing their health, increasing their understanding of IBD and, consequently, reducing the anxiety associated with this condition [79].

Similarly, at baseline (T0), there were no significant differences between the two groups regarding depression. At follow-up (T1), a significant difference was observed between Groups B and A. While both groups showed a significant improvement in depression, Group A showed a greater reduction compared to Group B, emphasizing how a personalized and targeted approach could be more effective in managing symptoms of depression. Effective treatment of depression is a healthcare priority [80], not only due to its high prevalence but also because it is one of the leading causes of disability worldwide [81]. An RCT study conducted by Salisbury et al. [82] to assess the impact of an integrated telemedicine service in caring for patients with chronic depression, compared to traditional care, demonstrated significant improvements in health in the patients involved. Additionally, a trial by Fortney et al. [83] demonstrated that a care model integrating communication technologies effectively managed depression, reducing severity and improving mental health status. In another RCT by Fortney et al. [84], the implementation of telehealth led to significant improvements in treatment-resistant depression in rural areas, suggesting particular efficacy in this context. The clinical course of IBD pathology has a significant impact on depression [21,85]. However, by assessing disease perceptions and implementing integrated care interventions, such as those explored in our study, we can achieve better management of depressive symptoms [86].

In terms of fatigue, no significant differences were observed between the two groups at baseline. At follow-up, there was a significant difference between Group B and A. Both Group A and Group B experienced a significant reduction in fatigue compared to baseline (Group A: median 56 vs. 50, *p* < 0.0001; Group B: median 57 vs. 54, *p* = 0.0136). This suggests that both groups benefited from a decrease in fatigue over the course of the study, although a more significant improvement was observed in Group A. Fatigue is one of the main symptoms of people with IBD [87,88,89], influenced by several factors, including treatment and lifestyle [90]. However, despite its impact on overall well-being [91], fatigue often receives suboptimal treatment in clinical practice. The study by Czuber-Dochan et al. [91] showed that healthcare professionals have a limited understanding of the impact of fatigue on the lives of IBD patients. This can be a barrier to accurate patient assessment. The review conducted by Galiano-Castillo et al. [92] highlights how remote support can mitigate the perception of fatigue. This underscores the importance of identifying and managing fatigue in patients with UC or CD to fully understand its impact and develop multimodal strategies to enhance their overall well-being [93].

Regarding satisfaction with participation in social life, at baseline (T0), no significant differences were observed between the two groups. At follow-up (T1), there was an improvement in social satisfaction in Group A compared to Group B (56.5 vs. 48.3, *p* < 0.0001). This indicates that the nursing intervention improved satisfaction with participation in social life in the group that received the personalized services compared to the control group. The debilitating symptoms of IBD, such as abdominal pain, diarrhea, fatigue, and sleep disturbance, can cause emotional and social distress, affecting their emotional well-being [94,95]. According to the findings of our study, literature has shown that the use of technology [96] for remote care delivery can successfully address social and educational challenges, thereby empowering IBD patients [96]. Furthermore, a randomized controlled trial conducted by Del Hoyo et al. [97], which incorporated a nurse-assisted telephone intervention, demonstrated an improvement in participation in social activities in IBD patients [98]. Therefore, the use of communication technologies to provide care and support to patients with inflammatory bowel disease, as highlighted by De Jong et al. [98], allows for personalized and timely interventions that also take into account psychosocial factors [98].

In the analysis of sleep quality, both groups showed significant improvements throughout the study. However, when the two groups were compared at follow-up, there was a significant difference in sleep quality, with Group A having better sleep quality than Group B (*p* = 0.0013). These results suggest that the intervention had a greater impact on promoting better sleep quality in Group A than in Group B. Previous studies have highlighted the complexity of sleep [99,100] and the importance of understanding the different components of sleep quality, through validated tools such as PSQI [101,102]. Research has identified a bidirectional relationship between poor-quality sleep and inflammatory bowel disease [103], suggesting that inadequate rest may contribute to increased bowel inflammation, exacerbating the condition [104,105]. As shown in the study by Xu et al. [106], access to ongoing nursing services improved the effect of treatment on patients, leading to improvements in physical condition and sleep quality. These findings align with those of our study, which indicated that nursing support, provided through a telephone call line or email assistance, led to enhanced sleep quality in Group A compared to Group B. The significant improvement observed in Group B could be due to many variables that influence sleep quality, such as diet [107], physical activity [108], access to insomnia medication, and other factors not yet studied [109,110].

Regarding quality of life, there was a significant improvement in Group A compared to Group B at 6 months (*p* < 0.05). This is particularly important, as quality of life is an important indicator for assessing the effectiveness of treatment in IBD patients [111]. In Group A, there was a statistically significant improvement in quality of life over time. In the control group, there was no significant difference in quality of life scores between T0 and T1. IBD patients are often exposed to high levels of stress and psychological distress [112], even during periods of remission of the disease [9]. It has been shown that high levels of psychological distress [113,114] are associated with an increased risk of disease exacerbations, as well as interference with social activities and relationships, and consequently with lifestyle [115]. Assessing the impact of IBD on quality of life is key to understanding the hidden burden that the disease imposes on affected individuals [9]. Improving the quality of life of people with IBD leads to improvements in emotional, social, and systemic functioning [116], as well as increased adherence [111,117,118]. In addition, previous studies have shown that targeted interventions, such as nursing support with easy access to resources and support services, can improve the quality of life of these patients [48,116,119]. The observed improvements in sleep quality and reduced fatigue go beyond mere numerical metrics; they reflect a heightened ability to manage daily activities, both physically and psychologically. Research supports the notion that such enhancements can foster better treatment adherence and decrease the risk of disease relapses [55,63]. For patients with chronic conditions such as IBD, these changes hold significant practical value, contributing to a more sustainable approach to disease management Notably, reductions in depression and fatigue also facilitate reintegration into social and professional activities, thereby improving the overall perception of quality of life. Addressing psychological well-being as a fundamental component of care is particularly critical, as depression, a common comorbidity in IBD, is well-documented to negatively impact disease progression and exacerbate its severity [120]. These findings underscore the importance of interpreting data not solely in terms of statistical significance but also in the context of their real-world implications for patients’ lives. The improvements observed in this study illustrate how personalized interventions can effectively reduce the physical and psychosocial burden of IBD, enhance overall well-being, and improve the efficiency of healthcare resource utilization.

The analysis of the results highlights the effectiveness of personalized nursing intervention in the context of IBD in promoting the psychological and social well-being of patients. This finding aligns with some findings in the scientific literature which show that integrated care with the support of remote communication interventions has led to improvements in the delivery of care to IBD patients, compared to unsupported interventions, even with non-unanimous results. It is necessary to consider the individual characteristics of patients, including their needs, preferences [121] and level of health literacy [122], to optimally tailor the interventions [48]. Importantly, the success of the intervention depends on several factors, including the quality of the nurse–patient relationship [123], the expertise of the nurses involved [124], and the availability of adequate resources [125]. Although the improvements observed in Group B were less pronounced than those in Group A, they nonetheless underscore the effectiveness of standard care in delivering meaningful clinical benefits. Specifically, the reduction in fatigue and the enhancement of sleep quality suggest that even traditional approaches, which include regular follow-ups and limited access to healthcare support, can positively impact the quality of life for IBD patients. These findings are consistent with prior studies demonstrating that continuous monitoring and access to information, even in the absence of structured interventions, can alleviate common symptoms such as fatigue and sleep disturbances [47,51]. The results from Group B provide a valuable baseline for evaluating the clinical benefits of standard care and highlight the importance of maintaining a consistent therapeutic approach. However, the comparison between the two groups emphasizes the added value of personalized interventions. Participants in Group A, who received tailored support through regular phone calls and email consultations with specialized nurses, reported significantly greater improvements in sleep quality, fatigue, and psychological parameters such as anxiety and depression. These findings reinforce the notion that personalized interventions not only enhance but also substantially augment the benefits of standard care in managing IBD [53,97].

Furthermore, the scientific literature has shown that telephone interventions are a viable choice for both patients and professionals, as they can reduce barriers to treatment and time to care [126,127]. For instance, some reviews have concluded that telephone interventions can improve quality of life and health outcomes for people with chronic diseases [128]. Other studies have shown that patients welcome phone calls from nurses for information, referrals, and emotional support [129,130]. The email service has also been evaluated as an effective communication tool between healthcare professionals and patients, as it can improve service efficiency, promote better continuity of care, reduce costs, and optimize patient outcomes [131,132,133]. The use of email can be a relevant tool to complement communication with patients, providing supplementary educational content, detailed explanations about their condition, answering follow-up questions, and optimizing appointment scheduling [134]. The most asked questions focused on aspects of daily life that have a significant impact on patients’ quality of life, such as sleep disturbance, fatigue, social interactions, and emotional distress. In a collaborative context within the intervention team, nurses’ responses to patients’ questions and needs were planned and shared. Therefore, nursing support appears to lead to better outcomes for IBD patients given their chronic characteristics and impact on physical and mental health.

This study provides preliminary results that need to be confirmed by a multicenter study and a large sample size. The choice of this preliminary study was intended to reduce the cost before performing a study on very large sample. It is important to recognize some limitations of our study. These include the small sample size due to the preliminary study and the reliance on data from a single health facility, which could compromise the generalizability of the results. In addition, the single-site design may not adequately reflect outcomes in diverse healthcare settings or systems, thereby limiting the external validity of our conclusions. To address this, future studies should aim for multicenter or international collaborations to better capture the variability in patient populations, healthcare practices, and system-level factors. Such studies would strengthen the robustness of the findings and enhance their applicability across different contexts. Additionally, we did not collect data on whether participants in the control group sought external support, such as additional medical consultations, psychological services, or peer support groups. This unmeasured variable may have influenced the outcomes, potentially biasing comparisons between the intervention and control groups. Future studies should consider systematically monitoring external support utilization to better understand its impact and account for it in the analysis. Another limitation is the relatively short follow-up period of six months, which may not fully capture the long-term effects of the nursing intervention. While our findings suggest meaningful improvements in key outcomes, such as anxiety, depression, fatigue, and sleep quality, it remains uncertain whether these benefits are sustained over time. Future studies should include longer follow-up periods to evaluate the durability of these effects and assess whether they translate into improved long-term health and quality of life for IBD patients. Furthermore, maintaining the unchanged disease status during the study period could influence the representativeness of our research concerning the wide variety of conditions present in IBD patients.

While the findings of our study highlight significant improvements in key outcomes, it is important to acknowledge that certain external factors were not controlled for in our analysis. For instance, patients’ adherence to medication, changes in treatment regimens, and access to additional healthcare services could have influenced the results. Variations in these factors may have contributed to the observed differences between the intervention and control groups. Future studies should incorporate methods to monitor and account for these external variables, such as tracking medication adherence, documenting treatment changes, and collecting detailed information on additional healthcare resource utilization. This approach would provide a more comprehensive understanding of the intervention’s effects and its interplay with other factors influencing patient outcomes.

Finally, it is essential to consider the resource implications of implementing programs on remote nursing interventions. Remote nursing interventions, including telephone and email support, require investments in personnel training, infrastructure, and time. However, these initial costs may be balanced by long-term benefits, such as reduced hospitalizations, fewer emergency visits, and improved medication adherence, which collectively lower overall healthcare costs. Previous studies have demonstrated that nurse-led interventions can achieve cost savings by improving patient outcomes and reducing the burden on healthcare systems [135,136]. Future research should incorporate detailed cost-effectiveness analyses to evaluate the sustainability and scalability of these interventions in diverse healthcare settings.

The integrated approach to caring for patients with IBD, combining personalized interventions with nursing support, represents a promising clinical practice that deserves further investigation and future development. In particular, the integration of innovative technologies and digital solutions could further enrich this approach, allowing more accurate patient monitoring, more efficient management of clinical data, and more effective communication between patients and healthcare professionals. The results of the study provide important insights for future research into IBD management. It would be useful to investigate the long-term effectiveness of similar interventions and assess their sustainability in the context of managing chronic diseases such as IBD. In addition, research into other factors that may influence quality of life, such as disease severity, adherence to treatment, and social support, could provide a more complete understanding of the effects of such interventions on the quality of life of IBD patients. This synergy between traditional care and technological tools could open up new perspectives on the treatment of IBD, improving the effectiveness of interventions and optimizing the overall patient experience.

## 5. Conclusions

The study showed that a nursing educational intervention, delivered through telephone support or email, can have a significant impact on the quality of life of IBD patients. The study findings underscore the critical role of personalized nursing interventions in improving the quality of life for patients with IBD. From a theoretical perspective, this research contributes to the growing body of literature emphasizing the psychosocial aspects of chronic disease management, particularly in relation to anxiety, depression, fatigue, and sleep quality. It also highlights the effectiveness of remote nursing interventions, filling a gap in the existing literature on the role of technology-mediated care in chronic disease management. Practically, the study provides actionable insights for healthcare providers, demonstrating that integrating remote nursing interventions into standard IBD care practices can significantly enhance patient outcomes. These findings support the adoption of such interventions as a cost-effective and scalable approach to improving patient well-being, particularly in resource-limited settings. Future research should explore strategies for broader implementation and evaluate their long-term sustainability.

## Figures and Tables

**Table 1:** Baseline socio-demographic characteristics.

Parameters	Group A Intervention	Group B Control	Group A vs. Group B	
	N = 26	N = 24	*p*-Value (Test)	Effect Size
Gender				
Males	53.8% (14)	54.2% (13)	0.98 (C)	phi = 0.003 trivial effect
Females	46.2% (12)	45.8% (11)		
Age				
18–45	65.4% (17)	54.2% (13)		phi = 0.13 small effect
46–69	30.8% (8)	37.5% (9)	0.65 (F)	
≥70	3.8% (1)	8.3% (2)		
Civil status				
Married	69.2% (18)	79.2% (19)	0.42 (C)	phi = 0.11 small effect
Unmarried	30.8% (8)	20.8% (5)		
Sons				
Yes	73.1% (19)	87.5% (21)	0.29 (F)	phi = 0.18 small effect
No	26.9% (7)	12.5% (3)		
Disease activity				
Remission	84.6% (22)	87.5% (21)	1.0 (F)	phi = 0.04 trivial effect
Acute	15.4% (4)	12.5% (3)		

C = chi-square test, F = Fisher’s exact test.

**Table 2:** Results of the PROMIS^®^ questionnaire.

PROMIS^®^	T0 Mean ± SD	T1 Mean ± SD	T0 vs. T1	
	Median (IRQ)	Median (IRQ)	*p*-Value (Test)	Effect Size
ANXIETY				
Group B (control) (n = 24)	67.7 ± 10.4	64.8 ± 10.6	<0.0001 * (W)	*r* = 0.86 large effect
	66 (61–74)	62 (58–72)		
Group A (nursing intervention) (n = 26)	65.4 ± 9.5	54.2 ± 6.3	<0.0001 * (W)	*r* = 0.84 large effect
	66 (62–68)	54 (50–60)		
**Group B vs. Group A**				
***p*-value (test)**	0.42 (Tu)	0.0001 * (Tu)		
**Effect size**	d = 0.01 trivial effect	d = 0.05 trivial effect		
DEPRESSION				
Group B (control) (n = 24)	57.0 ± 5.8	55.6 ± 6.8	0.0284 * (W)	*r* =0.81 large effect
	56 (52–61)	54 (50–61)		
Group A (nursing intervention) (n = 26)	54.8 ±3.2	50.1 ± 3.3	<0.0001 * (Tp)	d = 1.51 large effect
	56 (52–58)	50 (48–52)		
**Group B vs. Group A**				
***p*-value (test)**	0.11 (Tu)	0.0013 * (MW)		
**Effect size**	d = 0.02 trivial effect	*η*^2^ = 0.21 large effect		
FATIGUE				
Group B (control) (n = 24)	56.6 ± 4.1	55.3 ± 4.5	0.0136 * (W)	*r* =0.504 large effect
	57 (54–58)	54 (52–56)		
Group A (nursing intervention) (n = 26)	55.1 ± 3.8	51.2 ± 3.4	<0.0001 * (W)	*r* =0.83 large effect
	56 (52–58)	50 (48–54)		
**Group B vs. Group A**				
***p*-value (test)**	0.18 (Tu)	0.0010 * (MW)		
**Effect size**	d = 0.015 trivial effect	*η*^2^ = 0.221 large effect		
SATISFACTION, SOCIAL PARTICIPATION				
Group B (control) (n = 24)	58.8 ± 5.3	57.6 ± 6.1	0.11 (Tp)	d = 0.34 medium effect
	58 (55–63)	56 (52–62)		
Group A (nursing intervention) (n = 26)	56.5 ± 2.6	48.3 ± 3.7	<0.0001 * (Tp)	d = 2.6 large effect
	57 (54–58)	48 (46–50)		
**Group B vs. Group A**				
***p*-value (test)**	0.065 (Tu)	<0.0001 * (Tu)		
**Effect size**	d = 0.021 trivial effect	d = 0.074 trivial effect		

* = significant test, Tp = paired *t*-test, Tu = unpaired *t*-test, W = Wilcoxon test, SD = Standard deviation, IQR= interquartile range.

**Table 3:** Results of PSQI and IBDQ.

Pittsburgh Sleep Quality Index (PSQI) Sleep Quality	T0	T1	T0 vs. T1	T0 vs. T1
	Mean ± SD	Mean ±SD		
	Median (IRQ)	Median (IRQ)	*p*-Value (Test)	Effect Size
Group B (control) (n = 24)	4.9 ± 1.1	4.3 ± 0.9	0.0052 * (W)	*r* = 0.41 large effect
	5 (4–6)	4 (4–5)		
Group A (nursing intervention) (n = 26)	5.2 ± 0.8	3.2 ± 1.1	<0.0001 * (W)	*r* = 0.85 large effect
	5 (5–6)	3 (2–4)		
**Group B vs. Group A**				
***p*-value (test)**	0.34 (MW)	0.0013 * (MW)		
**Effect size**	*η*^2^ = 0.018 medium effect	*η*^2^ = 0.21 large effect		
Inflammatory Bowel Disease Questionnaire (IBDQ) Quality of life	**T0**	**T1**	**T0 vs. T1**	**T0 vs. T1**
	**mean ± SD**	**mean ± SD**		
	**Median (IRQ)**	**Median (IRQ)**	***p*-value (test)**	**Effect size**
Group B (control) (n = 24)	191.8 ± 5.6	193.0 ± 6.5	0.055 (W)	*r* = 0.17 medium effect
	192 (188–197)	193 (190–198)		
Group A (nursing intervention) (n = 26)	193.2 ± 3.9	199.8 ± 4.0	<0.0001 * (W)	*r* = 0.86 large effect
	192 (190–196)	199 (196–204)		
**Group B vs. Group A**				
***p*-value (test)**	0.31 (Tu)	0.0002 * (MW)		
**Effect size**	d = 0.012 small effect	*η*^2^ = 0.29 large effect		

* = significant test, Tu = unpaired *t*-test, Tp = p aired *t*-test, W = Wilcoxon test, MW = Mann–Whitney test, SD = Standard deviation, IQR= interquartile range.

## Data Availability

The raw data supporting the conclusions of this article will be made available by the authors on request (in order to respect the privacy of the participants).
